# Economic inequalities in decayed, missing, and filled first permanent molars among 8–12 years old Iranian schoolchildren

**DOI:** 10.1186/s12903-023-03471-4

**Published:** 2023-10-07

**Authors:** Maryam Khoramrooz, Seyed Mohammad Mirrezaie, Mohammad Hassan Emamian, Hajar Golbabaei Pasandi, Ali Dadgari, Hassan Hashemi, Akbar Fotouhi

**Affiliations:** 1 Department of Health Management and Economics, School of Public Health, Hamadan University of Medical Sciences, Hamadan, Iran; 2 Modeling of Noncommunicable Diseases Research Center, Hamadan University of Medical Sciences, Hamadan, Iran; 3 Center for Health Related Social and Behavioral Sciences Research, Shahroud University of Medical Sciences, Shahroud, Iran; 4 Ophthalmic Epidemiology Research Center, Shahroud University of Medical Sciences, Shahroud, Iran; 5 Student Research Committee, School of Public Health, Shahroud University of Medical Sciences, Shahroud, Iran; 6 School of Nursing and Midwifery, Shahroud University of Medical Sciences, Shahroud, Iran; 7 Noor Ophthalmology Research Center, Noor Eye Hospital, Tehran, Iran; 8 Department of Epidemiology and Biostatistics, School of Public Health, Tehran University of Medical Sciences, Tehran, Iran

**Keywords:** Economic inequality, Prevalence, First permanent molars, Dental caries, Schoolchildren, Iran

## Abstract

**Background:**

First permanent molars (FPM) play an important role in the masticatory function and oral health. This study aimed to assess the economic inequalities of FPM health indices among schoolchildren in the northeast of Iran.

**Methods:**

A total of 4051 children aged 8–12 years old were included in the analyses of this cross-sectional study in 2015. Economic status was measured using the principal component analysis on home assets. Concentration index (C) was used to measure economic inequality in FPM health indices, and its contributing factors determined by Wagstaff decomposition technique.

**Results:**

The prevalence of having decayed, missing, and filled FPMs among children was 40.9% (95% CI: 38.8–43.0), 1.2% (95% CI: 0.8–1.6%), and 7.8% (95% CI: 6.7–8.9%), respectively. Missing FPM was generally more concentrated among low-economic children (C=-0.158), whereas, filled FPM was more concentrated on high-economic children (C = 0.223). Economic status, mother education, having a housekeeper mother, and overweight/obesity, contributed to the measured inequality in missing FPM by 98.7%, 97.5%, 64.4%, and 11.2%, respectively. Furthermore, 88.9%, 24.1%, 14.5%, and 13.2% of filled FPM inequality was attributable to children’s economic status, father education, residence in rural areas, and age, respectively.

**Conclusion:**

There is a significant economic inequality in both missing and filled FPM. This inequality can be attributed to the economic status of individuals. To reduce FPM extraction, it is important to target low-income and rural children and provide them with FPM restoration services. Additionally, it is necessary to provide training to less-educated parents and housekeeper mothers to address the observed inequalities.

## Background

Dental caries is the most common non-communicable disease and an important public health problem worldwide that affects both children and adults [1, 2]. The prevalence of dental caries in children’s deciduous teeth in the world is 46.2% (95% CI: 41.6–50.8%), and 53.8% (95% CI: 50–57.5%) of children experience caries in their permanent teeth [3]. The results of a systematic review and meta-analysis study in Iran showed the overall prevalence of caries in deciduous and permanent teeth among Iranian children was 62.8% (95% CI: 52.2, 72.4%) and 78.6% (95% CI: 73, 83.2%), respectively [4].

The first permanent molars (FPMs) with the large occlusal surfaces, and multiple, strong and separate roots are considered as the most important unit of the mastication system [5], which usually erupt between the ages of 6 to 7 years [6]. These teeth play an important role in balanced occlusion and extraction of FPMs may cause deviations in dental arches and skeletal asymmetry [7]. Moreover, FPM is the most common permanent tooth in children that are prone to decay, because of the early eruption in the mouth, longer eruption time, and anatomical structure and position in the oral cavity [5]. It is also evident that caries in FPMs were associated with a number of carious lesions in other permanent teeth [8]. Parents’ socioeconomic status, diets with high carbohydrate, poor oral hygiene, caries experience in other teeth, and parents’ lack of knowledge on the FPM eruption time are the most common predisposing factors that can lead to FPM caries [8–13].

Treatment for oral health diseases is expensive and usually not supported by Universal Health Coverage (UHC) programs [14]. In low- and middle-income countries, the proportion of facing catastrophic health expenditures among households that incurred by dental health expenditures was much more than that of other households (35% vs. 7%) [15]. Oral diseases disproportionately affect the poor and socially disadvantaged people. Socioeconomic status, which is measured by income, occupation and education level, shows a strong association with the prevalence and severity of oral diseases. This association exists from early childhood to older ages and among all high-, middle-, and low-income countries [14].

The results of studies suggested that the prevalence of children’s FPM caries is high in different regions of Iran [16–19]. However, these studies have not been conducted with a large sample size. Moreover, the socioeconomic aspects of the FPM caries was not evaluated in these studies. This population-based study was designed to assess the prevalence and economic gradient of FPM health indices among 8-12-years-old schoolchildren in Shahroud, northeast of Iran. The results of this study can provide valuable information for policy-makers to design effective interventions to reduce FPM caries among schoolchildren and reduce health inequalities.

## Methods

### Source of data and study variables

Data for this cross-sectional study was extracted from the first phase of the Shahroud School Children Eye Cohort Study (SSCECS), which was run from February to September 2015. The Ethics Committee of Shahroud University of Medical Sciences approved the protocol of the study (Ethics registration number: 100/108,054). In this population-based study, which is one of the largest cohort studies in this age group in the world, in rural areas all eligible children were selected using the census method, and in the urban areas, a random cluster sampling method was used to select students from a total of 200 out of 473 classrooms as the clusters. Finally, 6624 students were selected, of which 5620 (84.8%) participated in the study. More details about objectives of SSCECS and its sampling method are provided elsewhere [20]. In all estimations, the sampling weights and the effect of cluster sampling are taken into account.

The sample size was calculated to accurately estimate DMFT and dft using the formula $n={(\frac{Z 1-\frac{\alpha }{2} \text{*} \sigma }{d})}^{2}$. In this formula, Alpha was considered equal to 0.05 and precisions of 0.02 for DMFT estimation and 0.1 for dft estimation were used. Previous studies in Iranian elementary school students [21] have reported a mean DMFT of 0.29 ± 0.93. The mean dft was reported as ranging from 2.90 ± 1.81 to 3.63 ± 1.71 [22, 23].

Using the results of these studies, the required sample size for DMFT estimation was 811 and for dft estimation, it ranged between 1114 and 1262. Considering the highest sample size and design effect equal to 2 (due to cluster sampling in urban areas), the final sample size was determined to be 2500 people. Due to the presence of other eye outcomes in SSCECS, 5620 students were examined, which is sufficient for the purposes of this study. Power analysis with PASS software was also used to ensure the sufficient sample size.

All participants in the first phase of SSCECS were included in this study and the only exclusion criterion was reluctance to have a dental examination. Dental examinations, including assessing tooth surfaces to diagnose tooth decay, were performed by two dentists with sufficient clinical experience [24]. The examination form was designed in accordance with the diagnostic methods and standards of the Oral Health Examination Survey published by the World Health Organization (WHO) [25]. For permanent teeth, the Decayed Missing Filled Teeth (DMFT) index was measured. D-component was evaluated as the any evidence of untreated or recurrent cavity in the tooth, M-component was recorded as the tooth extraction due to the caries, and F-component represents filled teeth without caries [24]. In addition, a questionnaire containing demographic and socioeconomic information, as well as, questions about home assets were filled out by the parents of the participating children.

Decayed FPM, Missing FPM, and Filled FPM were calculated using data from D-, M-, and F-components of FPM-DMFT index, respectively. Since frequency of FPM indices in the age groups of 6 and 7 years was low, only data for children in the age groups of 8–12 years were investigated.

The economic status was measured using the Principal Component Analysis (PCA) [26] on participants’ home assets including car, motorcycle, TV, LCD, bathroom, vacuum cleaner, washing machine, refrigerator, freezer, computer, telephone, internet, microwave, and dishwasher. The asset index is computed as the sum of the asset variables, weighted by the elements of the first eigenvector. This variable then categorized into five quintiles, indicating economic status of participants.

Demographic information including age, gender, place of residence (rural/urban), father’s education level (≤ 5, 5–12, and > 12), mother’s education level (≤ 5, 5–12, and > 12), father’s employment status (employed/unemployed or retired/other), mother’s employment status (housekeeper/other), type of school (public/private), overweight or obesity (yes/no), and having supplementary insurance (yes/no), were the other explanatory variables in the present study.

We used weight, height, sex and age data to calculate children’s Body Mass Index (BMI) for age Z-score (BAZ) according to the WHO 2007 Reference and AnthroPlus software [27, 28]. Children with a 1 < BAZ ≤ 2 were categorized as overweight. Obese children were those with a BAZ > 2.

### Measurement of inequality

The Concentration Index (C) [29] was measured to quantify the economic inequality in the distribution of the FPM health indices. This index was obtained from the concentration curve, which was plotted as the cumulative percentage of the FPM health indices against the cumulative percentage of children ranked by their economic status. The C is twice the area between the concentration curve and the line of equality (45-degree line) and can take values from − 1 to + 1. If the FPM health index is more concentrated among the low- (high-) economic children, the value of the C will be negative (positive), and the concentration curve is above (below) the line of equality. A concentration index of zero indicates equal distribution of health outcome.

The C can be written as follows:

1 $$\text{c}= 2\text{C}\text{o}\text{v}({\text{y}}_{\text{i}} , {\text{r}}_{\text{i}})/{\mu}$$

Where y_i_ is the FPM health index, µ is its mean, and r_i_ is the fractional rank of children i in the economic distribution. Since the outcome variables were binary, we used the conindex command in Stata to estimate a normalized C for the study outcomes using the Wagstaff approach [30, 31].

### Decomposition of inequality

The concentration indices (C) were decomposed to its contributors by applying the decomposition method proposed by Wagstaff et al. [29], as follows:

2 $$C={\sum }_{k}\left(\frac{{\beta }_{k}{\stackrel{-}{X}}_{k}}{\mu }\right){C}_{k}+\frac{{C}_{e}}{\mu }$$

In the above equation, ${\beta }_{k}$ is a vector of marginal effects of the study covariates which is obtained from the logit regression model. Then the marginal effects were multiplied by the mean of the covariates (${\stackrel{-}{X}}_{k}$) and divided by the mean of the outcome variable (µ). Finally, the contribution of each covariate to the measured inequality in the FPM health indices was calculated by multiplying the elasticity ($\frac{{\beta }_{k}{\stackrel{-}{X}}_{k}}{\mu }$) by the concentration index of the covariate (${C}_{k}$). The part of the measured inequality that cannot be explained by the study covariates was shown as the residual term ($\frac{{C}_{e}}{\mu }$).

## Results

Data required for this study was available for 4051 children. There were almost one-fifth of the studied children in each of the age groups from 8 to 11 years, and only the age group of 12 years had the lowest frequency of 553 (13.7%). Among the study participants, 2175 (54.2%) were boys, 3252 (90.6%) lived in urban areas, 2227 (55.3%) had a father with 6–12 years of education, 2284 (57.5%) had a mother with 6–12 years of education, 3486 (86.6%) had an employed father, 3383 (82.7%) had a housekeeper mother, 3749 (91.6%) attended public schools, 997 (25.7%) were overweight/obese, 785 (21.6%) covered by a supplementary insurance scheme (Table 1).

Mean FPM-DMFT was 0.97 ± 1.26. The mean of D-, M-, and F-components were 0.81 ± 1.16, 0.01 ± 0.15, and 0.14 ± 0.54, respectively. The prevalence of having decayed, missing, and filled FPM among children was 40.9% (95% CI: 38.8–43.0), 1.2% (95% CI: 0.8–1.6%) and 7.8% (95% CI: 6.7–8.9%), respectively.

Decayed FPM was more prevalent among the girls and older, non-obese/none-overweight children, and those with low-educated fathers and mothers, and from public schools. The prevalence of decayed FPM decreased from 43.1% in 1st economic quintile to 34.7% in 5th quintile. The prevalence of missing FPM increased with an increase in the age of children. Also, it was more prevalent among children who had less-educated and housekeeper mothers. Filled FPM was more prevalent among girls, urban children, and those who had supplemental insurance coverage and with high-educated fathers and mothers. Furthermore, it increased with increasing the age of children and an improvement in children’s economic status from 2.8% in the 1st quintile to 10.7% in the 5th quintile (Table 1).

**Table 1:** Prevalence of FPM health indices by demographic characteristics of students in Shahroud, Iran, 2015

Demographic characteristics		Total sample N (%)	Decayed FPM N (%)	Missing FPM N (%)	Filled FPM N (%)
Gender	Girl	1876 (45.76)	820 (43.34)	28 (1.42)	170 (9.86)
	Boy	2175 (54.24)	854 (38.85)	22 (1.01)	122 (6.00)
***P-value***			***0.031***	***0.323***	***< 0.001***
Age groups (years)	8	909 (22.28)	261 (28.37)	3 (0.38)	37 (4.34)
	9	959 (23.55)	375 (38.83)	7 (0.62)	51 (5.62)
	10	805 (19.74)	335 (41.00)	14 (1.65)	59 (7.76)
	11	825 (20.75)	404 (48.90)	13 (1.59)	79 (10.45)
	12	553 (13.68)	299 (52.64)	13 (2.29)	66 (12.97)
***P-value***			***< 0.001***	***0.006***	***< 0.001***
Place of residence	Urban	3252 (90.59)	1318 (40.53)	38 (1.17)	269 (8.27)
	Rural	799 (9.41)	356 (44.56)	12 (1.50)	23 (2.88)
***P-value***			***0.199***	***0.422***	***< 0.001***
Father education (years)	0–5	834 (17.80)	397 (47.59)	14 (1.83)	32 (4.65)
	6–12	2227 (55.30)	937 (42.00)	30 (1.28)	153 (7.24)
	> 12	990 (26.90)	340 (34.25)	6 (0.62)	107 (10.90)
***P-value***			***< 0.001***	***0.117***	***< 0.001***
Mother education (years)	0–5	1006 (21.56)	453 (45.03)	18 (2.03)	51 (6.07)
	6–12	2284 (57.53)	965 (42.11)	30 (1.23)	158 (7.29)
	> 12	761 (20.91)	256 (33.34)	2 (0.27)	83 (10.83)
***P-value***			***< 0.001***	***0.024***	***< 0.001***
Father employment status	Employed	3486 (86.59)	1427 (40.65)	41 (1.13)	254 (7.82)
	Unemployed/retired	397 (9.52)	171 (42.29)	5 (1.29)	30 (8.44)
	Other	168 (3.89)	76 (45.31)	4 (2.45)	8 (4.90)
***P-value***			***0.492***	***0.365***	***0.477***
Mother employment status	Housekeeper	3383 (82.73)	1419 (41.54)	45 (1.34)	232 (7.44)
	Other	668 (17.27)	255 (37.87)	5 (0.53)	60 (9.30)
***P-value***			***0.093***	***0.049***	***0.107***
Economic status quintiles	Poorest	727 (14.45)	307 (43.06)	10 (1.59)	19 (2.77)
	Poorer	783 (18.47)	347 (43.46)	11 (1.31)	39 (5.45)
	Medium	874 (22.24)	375 (42.63)	13 (1.41)	64 (7.73)
	Wealthier	820 (21.72)	351 (42.21)	11 (1.26)	80 (9.97)
	Wealthiest	847 (23.13)	294 (34.65)	5 (0.60)	90 (10.70)
***P-value***			***0.001***	***0.391***	***< 0.001***
Type of school	Public	3749 (91.59)	1575 (41.65)	46 (1.19)	264 (7.63)
	Private	302 (8.41)	99 (32.78)	4 (1.32)	28 (9.27)
***P-value***			***0.022***	***0.821***	***0.427***
Overweight/obesity	No	3054 (74.27)	1307 (42.44)	42 (1.34)	218 (7.74)
	Yes	997 (25.73)	367 (36.48)	8 (0.80)	74 (7.82)
***P-value***			***0.002***	***0.151***	***0.935***
Supplemental insurance	No	3266 (78.39)	1360 (41.15)	41 (1.23)	216 (7.22)
	Yes	785 (21.61)	314 (40.03)	9 (1.09)	76 (9.72)
***P-value***			***0.595***	***0.746***	***0.035***

The results of power analysis for the association between economic status and the study outcomes in the logistic regression models, showed that a sample size of 4051 observations achieves 99.7%, 97.6% and 98.3% power at a 0.05 significance level for decayed, filled and missing teeth, respectively. According to the results of the multiple logistic regression analysis in Table 2, boys were less likely to have a decayed FPM and filled FPM than girls. The odds of having decayed FPM, missing FPM, and filled FPM were increased with an increase in the children’s age. Rural children were less likely to have filled FPM than the urban children. The odds of having filled FPM was increased with an improvement in children’s economic status. Overweight/obese children were less likely to have decayed FPM.

**Table 2:** Association of FPM health indices with demographic characteristics of children in multiple logistic regression, Shahroud, Iran, 2015

Independent variables		Adjusted Odds Ratio (95% CI)		
		Decayed FPM	Missing FPM	Filled FPM
Male gender		0.85 (0.74, 0.98)^*^	0.72 (0.37, 1.39)	0.55 (0.43, 0.71)^*^
Residence in rural areas		1.01 (0.79, 1.28)	0.90 (0.40, 2.01)	0.50 (0.31, 0.83)^*^
Attend a private school		0.84 (0.62, 1.14)	2.26 (0.82, 6.23)	0.88 (0.55, 1.41)
Overweight/obesity		0.77 (0.66, 0.91)^*^	0.59 (0.30, 1.18)	0.88 (0.67, 1.15)
Have supplemental insurance		1.08 (0.91, 1.28)	1.17 (0.53, 2.57)	1.00 (0.75, 1.33)
Age groups	8	Reference group	Reference group	Reference group
	9	1.61 (1.27, 2.03)^*^	1.74 (0.46, 6.66)	1.35 (0.87, 2.11)
	10	1.83 (1.46, 2.29)^*^	4.77 (1.47, 15.48)^*^	1.97 (1.29, 3.00)^*^
	11	2.56 (2.05, 3.20)^*^	4.80 (1.42, 16.20)^*^	2.54 (1.71, 3.79)^*^
	12	2.93 (2.30, 3.75)^*^	6.40 (1.88, 21.78)^*^	3.38 (2.17, 5.26)^*^
Father education (years)	0–5	Reference group	Reference group	Reference group
	6–12	0.87 (0.70, 1.07)	1.00 (0.45, 2.23)	1.45 (0.91, 2.32)
	> 12	0.73 (0.56, 0.94)^*^	0.83 (0.22, 3.15)	1.96 (1.14, 3.36)^*^
Mother education (years)	0–5	Reference group	Reference group	Reference group
	6–12	1.01 (0.84, 1.21)	0.64 (0.29, 1.44)	0.84 (0.58, 1.21)
	> 12	0.84 (0.64, 1.11)	0.19 (0.03, 1.34)	1.02 (0.64, 1.63)
Father employment status	Employed	Reference group	Reference group	Reference group
	Unemployed/Retired	0.95 (0.74, 1.22)	0.89 (0.31, 2.54)	1.09 (0.69, 1.47)
	Other	1.09 (0.77, 1.55)	1.92 (0.57, 6.48)	0.75 (0.30, 1.85)
Mother employment status	Other	Reference group	Reference group	Reference group
	Housekeeper	1.02 (0.84, 1.25)	0.62 (0.20, 1.91)	0.99 (0.72, 1.37)
Economic status quintiles	Poorest	Reference group	Reference group	Reference group
	Poorer	1.07 (0.85, 1.36)	0.95 (0.38, 2.39)	1.69 (0.93, 3.04)
	Medium	1.02 (0.82, 1.28)	0.99 (0.42, 2.32)	2.22 (1.24, 3.95)^*^
	Wealthier	1.05 (0.82, 1.35)	0.95 (0.42, 2.13)	2.72 (1.54, 4.79)^*^
	Wealthiest	0.80 (0.61, 1.03)	0.50 (0.18, 1.38)	2.63 (1.49, 4.64)^*^

^* P<0.05; CI: Confidence Interval^

**Fig. 1:**
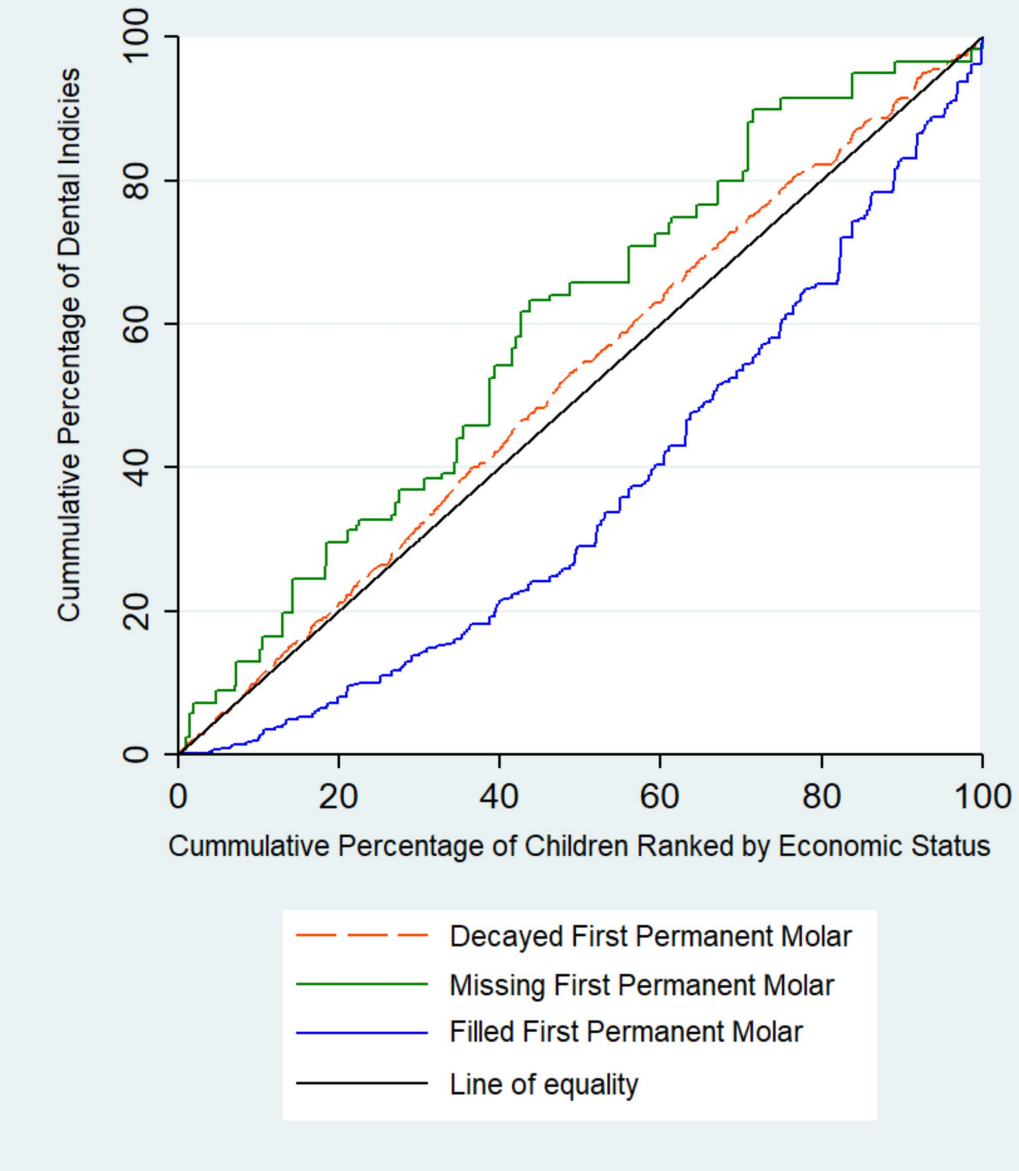
Concentration curves for first permanent molar (FPM) health indices among schoolchildren in Shahroud, Iran, 2015

As shown in Table 3; Fig. 1, the negative C of missing FPM (-0.158) was statistically significant and its concentration curve lies above the line of inequality, indicating that it was generally more concentrated among low-economic children. The positive and statistically significant C for filled FPM (0.223) and lying its concentration curve below the line of equality indicates that filled FPM was more concentrated among high-economic children. The negative C for decayed FPM (-0.065) was small and statistically significant, suggesting that it was more concentrated among low-economic children. However, the observed inequality was not outstanding.

**Table 3:** Descriptive statistics and concentration index for first permanent molar teeth (FPM) health indices among schoolchildren in Shahroud, Iran, 2015

FPM health indices	N (n)	Prevalence	95% CI	Concentration index	95% CI	P-value
**Decayed** **FPM**	4051 (1674)	40.91	(38.83, 42.99)	-0.065	(-0.086, -0.044)	0.003
**Missing FPM**	4051 (50)	1.20	(0.79, 1.61)	-0.158	(-0.235, -0.081)	0.041
**Filled FPM**	4051 (292)	7.76	(6.65, 8.88)	0.223	(0.185, 0.261)	< 0.001

^†^CI: Confidence Interval

Results of decomposition analysis for the observed inequalities in the missing FPM and filled FPM are presented in Table 4. According to the results, economic status and mother education were the two main factors that positively contributed to the measured inequality in missing FPM by 98.7% and 97.5%, respectively, followed by having a housekeeper mother and being overweight/obese with the positive contributions of 64.4% and 11.2%, respectively. Also, age and attending private schools decreased this inequality by 32.1% and 21.9%, respectively. Regarding the measured inequality in filled FPM, 88.9% of the inequality was attributable to the children’s economic status. Also, father education, residence in rural areas, and age were the next factors with the positive contributions of 24.1%, 14.5%, and 13.2%, respectively.

**Table 4:** Decomposition of economic inequalities in first permanent molar (FPM) health indices in Shahroud, Iran, 2015

Independent variables		Marginal effect		Elasticity		C_k_	Absolute contribution to CI		% Contribution	
		Missing FPM	Filled FPM	Missing FPM	Filled FPM		Missing FPM	Filled FPM	Missing FPM	Filled FPM
Male gender		-0.004	-0.041	-0.178	-0.285	0.073	-0.013	-0.021	8.2	-9.3
Age group (RC: 8-years-old)	9	0.007	0.021	0.128	0.063	-0.083	-0.011	-0.005	6.7	-2.4
	10	0.018	0.047	0.301	0.119	0.006	0.002	0.001	-1.1	0.3
	11	0.018	0.065	0.318	0.172	0.093	0.030	0.016	-18.7	7.2
	12	0.022	0.084	0.248	0.148	0.121	0.030	0.018	-19.0	8.1
	Sum						0.051	0.029	-32.1	13.2
Residence in rural areas		-0.001	-0.047	-0.010	-0.057	-0.562	0.005	0.032	-3.4	14.5
Father education (RC: 0–5 years)	6–12	0.317*10^− 4^	0.026	-0.001	0.183	-0.112	0.163*10^− 3^	-0.020	-0.1	-9.2
	> 12	-0.002	0.046	-0.049	0.161	0.461	-0.023	0.074	14.2	33.3
	Sum						-0.022	0.054	14.1	24.1
Mother education (RC: 0–5 years)	6–12	-0.005	-0.012	-0.249	-0.091	-0.043	0.011	0.004	-6.8	1.8
	> 12	-0.019	0.002	-0.334	0.004	0.495	-0.165	0.002	104.3	0.9
	Sum						-0.154	0.006	97.5	2.7
Father employment status (RC = Employed)	Unemployed/Retired	-0.001	0.006	-0.011	0.008	-0.063	0.001	-0.483*10^− 3^	-0.4	-0.2
	Other	0.008	-0.020	0.025	-0.010	-0.281	-0.007	0.003	4.4	1.3
	Sum						-0.006	0.002	4.0	1.07
Have a housekeeper mother		0.006	0.001	0.391	0.008	-0.261	-0.102	-0.002	64.4	-0.9
Economic status (RC = Poorest)	Poorer	-0.001	0.036	-0.009	0.086	-0.646	0.006	-0.055	-3.7	-24.9
	Medium	-0.164*10 − 3	0.055	-0.003	0.157	-0.153	0.468*10^− 3^	-0.024	-0.3	-10.8
	Wealthier	-0.001	0.069	-0.011	0.193	0.409	-0.004	0.079	2.8	35.4
	Wealthiest	-0.008	0.067	-0.158	0.199	1.000	-0.158	0.199	100.0	89.2
	Sum						-0.156	0.198	98.7	88.9
Attend a private school		0.010	-0.009	0.067	-0.009	0.518	0.035	-0.005	-21.9	-2.2
Overweight / obesity		-0.006	-0.009	-0.132	-0.029	0.135	-0.018	-0.004	11.2	-1.7
Have supplemental insurance		0.002	0.767*10^− 5^	0.033	0.214*10^− 4^	0.255	0.008	0.544*10^− 5^	-5.3	0.002
**Total observed**							**-0.373**	**0.291**	**235.4**	**130.3**
**Residual**							**0.214**	**-0.068**	**-135.4**	**-30.3**
**Total**							**-0.158**	**0.223**	**100**	**100**

RC: Reference Category

## Discussion

This study attempted to assess the prevalence and economic gradient of FPM health indices among 8–12 years old schoolchildren in northeast of Iran. In consistence with the findings of other studies [16, 19], the results of this study showed much higher prevalence of decayed FPMs (40.9%) than the filled FPMs (7.8%), which implies the children’s unmet needs for restoration services. Furthermore, although a small percentage (2%) of children had missing FPM, there is an urgent need for their dental implant due to the critical role of FPM in masticatory function and oral health [6–8].

Logistic regression results showed that the odds of having a decayed FPM in boys is less than the girls. Results of studies conducted in two cities of Iran [16, 19], indicated similar findings. However, some studies have not shown a difference in decayed FPM between the two genders [18, 32]. Although girls have more adherence to dental self-care recommendations [16], factors such as earlier eruption of FPM, different FPM anatomy, and earlier puberty may increase their risk of FPM caries [33, 34]. Our results also showed higher odds of filled FPM among the girls. Other studies in Iran have shown similar [18, 32] and opposite [16, 19] findings. More FPM restoration among the girls could be due to their more FPM caries [16] and/or more importance given to the beauty and attention of the parents to oral health of their girls. In consistence with other studies [35–37], it was found that children with higher ages are more commonly affected by FPM caries. Results of the present study also indicated obese/overweight children were less likely to have decayed FPM. Other studies have shown similar [38, 39] and opposite findings [40]. Also, in our study, the C of obesity/overweight was 0.135 (Table 4), which reveals the co-existence of children’s obesity/overweight and higher socioeconomic status (SES) [41–43]. Earlier eruption of FPMs in children with higher body mass index [33] and acidification of saliva and saliva flow among obese/overweight children can increase their risk of dental caries [44]. However, it seems that the parents of high-economic obese/overweight children had more awareness and facilities to observe their oral hygiene, treat their decayed teeth [10, 45–47], and also, follow a standard breastfeeding program which can prevent the occurrence of Early Childhood Caries (ECC) in children [48]. On the other hand, tooth decay can lead to toothache, decreased appetite and weight loss among low-economic non-obese/overweight children [43].

Among socioeconomic indicators, rural residency, having a more-educated father, and having higher economic status have increased the odds of FPM restoration. Children of more-educated fathers had less unmet needs for restoration of their carious FPM, as well. Other studies have suggested that the prevalence and severity of FPM caries is higher among rural than the urban children [17, 49] and children of less-educated fathers [50]. Dental caries and its extraction were more prevalent among low-SES children [51], whereas, high-SES children were more likely to fill their carious teeth [52]. It seems that high-SES parents have more awareness and facilities to observe children’s oral hygiene and treat their decayed teeth [10, 45–47].

In addition to the prevalence of FPM health indices, their distribution among the children with different economic status is also of particular importance. The concentration of decayed FPMs was slightly higher among the low-economic children, however, the difference was not considerable. One possible explanation for the small size of the inequality in decayed FMPs could be that high-economic families are more financially capable of providing their children with access to toothbrushes, toothpaste, floss, and dental check-up services to prevent their FPM caries [53, 54]. However, the financial advantage enjoyed by such households may also lead to their children consuming foods with a high calorie and sugar content, which can contribute to tooth decay [55].

We also found missing FPM was more concentrated among poor children, whereas, filled FPM was more concentrated among the high-economic children. According to the results of the decomposition analyses, the measured inequalities in carious FPM extraction and restoration was largely explained by the households’ economic status, indicating that health interventions on poor children can greatly reduce these inequalities. Additionally, having a less-educated and a housekeeper mother, which were more prevalent among the poor children, have increased the inequality in missing FPM, and father’s education had positive contributions on the inequality in the treatment outcomes (extraction and restoration) of FPM caries. It seems that less-educated parents and housekeeper mothers have less awareness and income to support their children with an on-time prevention and treatment of carious teeth, including FPMs [45–47].

Residence in rural areas was the next factor that had positive contribution to the measured inequality in children’s FPM restoration. Other studies have shown more dental caries and less filled teeth among rural children [56, 57]. In our study, although there was no significant difference in the odds of decayed FPM between rural and urban children, rural children who were commonly poor, were less likely to utilize dental filling services. Financial and physical access to dental restoration services may be among the factors that could explain less utilization of FPM restoration services among poor rural children [58].

Age had a negative contribution to the more concentration of missing FPM among the low-economic children, and a positive role to the more concentration of filled FPM among high-economic children. It can be concluded that younger children, who were commonly from lower economic quintiles, have a higher priority to take interventions to improve their dental hygiene and benefit from timely FPM restoration services.

The study results also indicated attending private schools decreased the measured inequality in children’s missing teeth. The results of our study indicated although children in private schools have less FPM caries, their FPM extraction was more than those from public schools. More teeth extraction among children attending private schools was also reported in other studies [59, 60]. These findings show that private school children, who are mainly from the high-economic groups, have better dental self-care behaviors [59]. However, one reason for the more FPM extraction among these children could be that their high-economic households have more financial ability to buy cariogenic food and drinks, which regular consumption can increase the severity of FPM caries and the probability of its extraction [59]. On the other hand, lower FPM extraction among low-economic children attending public schools could be due to delaying extraction of the carious FPM that may arise from lack of parental awareness and/or financial access to dental health services.

This study was the first one in Iran in which the economic inequalities in FPM health indices was assessed. The study findings provide evidences for planning interventions to reduce economic inequalities in FPM health indices. However, it has some limitations, as follows: (1) We have no data on children’s dental self-care and dietary habits, utilization of dental health services, including checkups, parents’ and children’s awareness about FPM and its hygiene. This issue caused high residual component of the measured inequality in missing FPM (-135.4%). Further investigations could be performed to explain the prevalence and economic gradients in FPM health indices with factors related to their awareness and performance. (2) Despite recruiting a large sample in size, the results of this study cannot be generalized to all Iranian schoolchildren, because it did not include all the parts of the country. (3) Demographic data have been collected through a self-administered questionnaire. (4) The inter-examiner reliability of dental examinations has not been evaluated, however, at the beginning of the study, to ensure an identical performance in detecting dental caries, dentists were explained by the procedure of dental examinations and recording the results. (5) Because of the cross-sectional design of the study, casual interpretations should be done with caution.

## Conclusions

The results of the present study revealed a significant economic inequality in the treatment outcomes of children’s FPM caries, such that FPM extraction was more concentrated among low-economic children and FPM restoration was more concentrated among the high-economic children. Economic status was the main factor that explained these inequalities. Therefore, low-economic and rural children should be targeted in government interventions to reduce economic inequality in FPM health indices. Furthermore, training less-educated parents and housekeeper mothers on the dental health, and increasing their access to dental health services are the other interventions that could largely decrease the observed inequalities in the treatment outcomes of FPM caries.

## Data Availability

The data that support the findings of this study are available from the corresponding author upon a reasonable request.

## Abbreviations

FPM: First Permanent Molar
UHC: Universal Health Coverage
SSCECS: Shahroud School Children Eye Cohort Study
WHO: World Health Organization
DMFT: Decayed, Missing and Filling Teeth
PCA: Principal Component Analysis
BMI: Body Mass Index
BAZ: BMI for Age Z-score
C: Concentration Index
Cov: Covariance
CI: Confidence Intervals
RC: Reference Category
SES: Socioeconomic Status
